# Abdominal radical trachelectomy guided by sentinel lymph node biopsy for stage IB1 cervical cancer with tumors >2 cm

**DOI:** 10.18632/oncotarget.13788

**Published:** 2016-12-03

**Authors:** Xiangyun Deng, Ying Zhang, Dapeng Li, Xiaoling Zhang, Hui Guo, Fei Wang, Xiugui Sheng

**Affiliations:** ^1^ School of Medicine and Life Sciences, University of Jinan, Jinan 250022, Shandong, People's Republic of China; ^2^ Shandong Academy of Medical Sciences, Jinan 250000, Shandong, People's Republic of China; ^3^ Shandong Cancer Hospital Affiliated to Shandong University, Jinan 250117, Shandong, People's Republic of China

**Keywords:** cervical cancer, tumor >2 cm, abdominal radical trachelectomy, sentinel lymph node, oncological outcomes

## Abstract

Accuracy of prediction of pelvic lymph node status using sentinel lymph node biopsy (SLNB), and outcomes of SLNB-guided abdominal radical trachelectomy (ART) were assessed. Patients with stage IB1 (Figure 2009) cervical cancer and with tumors >2 cm were enrolled. Prior to fertility-sparing surgery 99mTc-labeled phytate was administered. SLNs were intraoperatively identified, excised, and assessed using fast-frozen sections. Systematic bilateral pelvic lymphadenectomy with or without para-aortic lymphadenectomy was subsequently undertaken. The SLN detection rate was 91.8% (45/49 patients); 8.2% (4/49) had radical hysterectomies because of metastatic primary SLNs. All SLNs received routine immunopathological examination to detect micrometastasis. Sensitivity, accuracy, and false negative rates were 100%, 100%, and 0%, respectively. ART was successfully completed in 45 patients (median follow-up, 61 [range, 4–149] months). Three of the 45 (6.7%) were lost to follow-up; two relapsed and one died of tumor progression. Overall 3-year survival and progression-free survival rates were 97.6% and 95.2%, respectively. Of the 19 patients who attempted to conceive after surgery, five achieved pregnancy, and one had a live birth in the third trimester. We concluded that SLNB using 99mTc-labeled phytate can accurately assess pelvic node status. SLNB-guided ART is safe and feasible in patients selected for fertility-sparing procedures.

## INTRODUCTION

Cervical cancer is the second most common gynecological malignancy, and the number of patients in their childbearing years that are diagnosed with the early stages of this cancer is increasing [1]. Preserving fertility is a common desire for young cervical cancer patients. When evaluating preservation of the uterus, tumor size is something to take into consideration. Larger lesions suggest an increased risk of recurrence [2, 3]. The 2015 National Comprehensive Cancer Network (NCCN) cervix cancer guidelines states that radial trachelectomy and pelvic lymph node (PLN) dissection, with or without para-aortic lymph node sampling, is an option for patients with stage IB1 disease that want to sustain their fertility. This is usually only performed in patients with tumors 2 cm in size or smaller. Tumors >2 cm in size are left to the surgeon's discretion. Some surgeons suggest that a 2-cm cutoff should be used for a vaginal trachelectomy and a 4-cm cutoff for an abdominal trachelectomy (e.g., laparotomy, laparoscopic and robotic) [4].

A major indicator in cervical cancer patients is PLN status; it affects clinical outcomes and decisions regarding treatment [5]. In stage IB cervical cancer, the prevalence of PLN metastasis has been estimated to be approximately 16.7% [6]. This means that the majority of early cervical cancer patients who undergo pelvic lymph node dissection will not gain any benefit from the procedure. They will also be subjected to a considerable number of complications such as lymphedema, lymphocyst, and vessel or nerve damage [7].

Sentinel lymph node (SLN) mapping is becoming more prominent in the management of cervical cancer. SLN mapping aims to identify cervical cancer patients who are SLN negative for metastases by using frozen sections; this approach avoids complete pelvic lymphadenectomy [8, 9]. SLN mapping is also used for the selection of candidates for fertility sparing treatment in young patients [10–12]. Abdominal radical trachelectomy (ART) is a possible application of SLN mapping in patients who want to preserve fertility. However, it is unclear if it would be safe to use SLN biopsy (SLNB) intro-operatively to select candidates for ART among stage IB1 patients that have tumors larger than 2 cm.

To assess the feasibility and accuracy of the SLNB procedure in this study, we used 99mTc-labeled phytate to guide ART in patients with stage IB1 cervical cancer that had tumors larger than 2 cm in size. We also evaluated the safety of SNLB-guided ART by observing surgical, oncologic and fertility outcomes in patients who wished to preserve their fertility.

## RESULTS

Forty-nine patients were identified and entered into the study group. Patient characteristics are summarized in Table 1.

**Table 1:** Characteristics of the 49 patients in our study

Characteristics	No. of patients
Mean age (range)	28.5 years (19-40)
Childbearing history	
0	20
≥1	29
Histology	
Squamous cell carcinoma	46
Adenocarcinoma	3
Tumor size	
2 cm < Tumor size < 3 cm	20
3 cm ≤ Tumor size ≤ 4 cm	29
Cell differentiation	
Well	17
Moderate	17
Poor	15
Total	49

### Procedure details and complications

The proportion of patients who underwent ART was 91.8% (45/49); the remaining 8.2% (4/49) had radical hysterectomies as a result of positive primary SLNs. The median surgical time was 235 (range, 175–280) min; median blood loss was 450 (range, 300–1000) ml. Intraoperative complications included bladder muscle-layer injury in one of 49 (2.0%) patients. The patient with bladder muscle-layer injury received urethral catheterization for 2 weeks and bladder function finally recovered postoperatively. Postoperative complications included pelvic lymphocysts in seven of 49 (14.3%) patients and post-trachelectomy stenosis of the neo-cervix in three of 49 (6.1%) patients.

### SLN detection results

**Figure 1:**
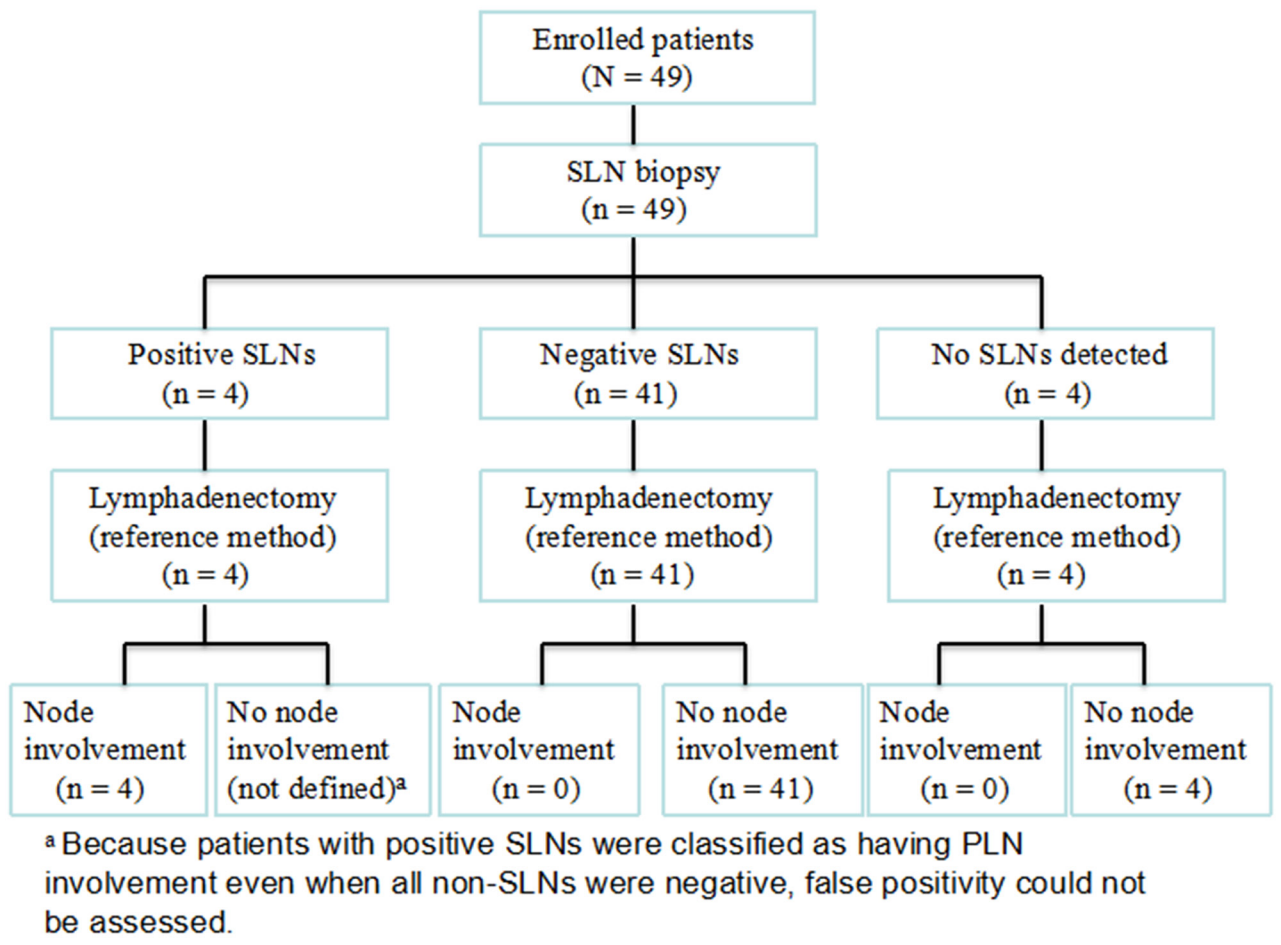
Outcome of sentinel lymph node biopsy in 49 patients with stage IB1 cervical cancer and tumors >2 cm in size

We detected 1008 PLNs, including 145 SLNs in the population of 45 patients. The SLN detection rate was 91.8% (45/49) and the median number of SLNs identified per patient was 3.2. The obturator was the most common location for SLN detection, followed by the external and internal iliac and the common iliac (Table 2). Bilateral pelvic SLNs were detected in 69.4% (34/49) of patients. Intraoperatively, the SLNs of four (8.9%) patients were found to be positive on frozen sections; they then underwent immediate complete radical hysterectomies. In the other 41 (91.1%) patients, the intraoperative frozen SLN sections and bilateral ovarian biopsies were negative and ART was successfully completed. The results for the frozen sections and the permanent staining were identical. A routine pathologic examination revealed that all four patients with positive SLNs had metastatic PLNs. In this examination there were no false negative SLNs and there were no cases of metastasis in the non- SLNs (Figure 1). The sensitivity and accuracy rates were both 100%, and the false negative rate was 0%; the negative predictive value of the SLNs was 100% as well (Table 3). Results of anti-keratinose immunohistochemical staining indicated no missed micrometastases (lesions <2 mm) or isolated tumor cells (ITCs) in SLNs (data not shown).

**Table 2:** Localization and status of the SLNs

Details of SLNs	No. of patients (%)
SLNs detection	45/49 (93.8%)
Total number of SLNs	145
Localization of SLNs	
Common iliac	8/145 (5.5%)
External and internal iliac	46/145 (31.7%)
Sacral	1/145 (0.7%)
Obturator	89/145 (61.4%)
Para-aortic	1/145 (0.7%)
Latus of pelvic SLNs	
Unilateral	11/49 (22.4%)
Bilateral	34/49 (69.4%)

**Table 3:** Diagnosis of lymph node involvement using SLNB, compared with full lymphadenectomy, in patients with stage IB1 cervical cancer with tumors > 2 cm

Metastatic lymph node diagnosis^a^	Lymphadenectomy		
	Yes	No	Total
SLN biopsy^b^			
Yes	4	0	4
No	0	41	41
Total	4	41	45

a Presence of macrometastasis, micrometastasis, and/or ITC.

b Sensitivity: 100.0% (95% CI: 47.3–100.0%); accuracy: 100.0% (95% CI, 92.9–100.0%);

positive predictive value: 100.0% (95% CI: 47.3–100.0%); negative predictive value: 100.0% (95% CI: 92.9–100.0%).

### Adjuvant therapy

In 45 (91.8%) patients who underwent ART, 16 (35.6%) received adjuvant therapy. Two of the patients showed three intermediate risk factors: poor differentiation, deep stromal invasion (DSI), and lymphovascular space invasion (LVSI). These patients were treated with concurrent chemotherapy. Chemotherapy treatment was prompted by the occurrence of any two intermediate risk factors in six patients and only one intermediate risk factor in eight patients. Of these patients, eight received chemotherapy involving combination PVB, five received PEB chemotherapy, and three adenocarcinoma patients received TC chemotherapy. Four patients who underwent radical hysterectomy received concurrent chemo-radiotherapy.

### Outcomes of follow-up

ART was successfully completed in 45 patients with a median follow-up time of 61 months; the range of the follow-up time was 4-149 months. By July 2015, three of the 45 (6.7%) patients had been lost, leading to a follow-up rate of 93.3%. Of the three patients lost to follow-up, one was out of contact and the other two gave up reexaminations. They did not differ significantly from the patients remaining in the study in terms of age (P=0.215), tumor size (*P* = 0.147), preoperative conization (*P* = 0.437) or complications (*P* = 0.279). Of the remaining 42 patients, two (4.8%) suffered relapses with pelvic recurrences at 28 and 30 months after their initial diagnoses; they received four cycles and six cycles of combination PEB, respectively. One patient died of tumor progression at 34 months. The characteristics of the two patients with recurrence are summarized in Table 4. The other 40 patients were progression-free during the follow-up period. Their overall survival and progression-free survival rates at 3 years were 97.6% and 95.2%, respectively.

**Table 4:** Characteristics of patients who suffered recurrences

No.	Histology	Tumor size	Cell differentiation	Deep stromal invasion	Lymphovascular space invasion	Progression-free survival (months)	Adjuvant therapy	Result
1	Squamous	3cm	Poor	< 1/2	Positive	28	6 cycles of combination PEB	Death
2	Squamous	2.5cm	Poor	<1/2	Negative	30	4 cycles of combination PEB	Alive

Of the 19 patients who wished to conceive, five succeeded, for a total of five pregnancies after surgery. Of these five pregnancies, one was a term delivery, one was a mid-trimester miscarriage and three were first-trimester miscarriages. The newborn developed well.

## DISCUSSION

The results of the latest systematic review show the pooled detection rate of SLN mapping in early stage cervical cancers is 89.2% [7]. Although it has been reported that a larger tumor size (>2 cm) and a more advanced disease stage (>IB2) are associated with low detection rates and sensitivity [7, 13], a comparison of the subgroups regarding tumor size has not generally been pre-specified in the study protocol; hence, the reported results might be influenced by differences in disease stage and the prevalence of metastatic disease. The present study was focused on stage IB1 patients with tumors >2 cm in size. The results of the study suggest that SLN mapping may also be a sensitive method for detecting node metastases on stage IB1 cervical cancer patients under the conditions of tumor size being greater than 2 cm.

Lecuru et al. demonstrated that when the identification is bilateral, a negative SLN accurately predicts the absence of pelvic node involvement in early cervical cancer patients [5, 14]. The reported bilateral SLN detection rates were between 24% and 88%, as compared with 69.4% in our series [15–17]. In the current study, a higher number of bilateral nodes was detected than in our previous study. This may suggest that the rate of SLN identification will improve significantly with time and experience. Because intracervical injection is technically more difficult, the learning curve effect for cervical cancer is likely to be in the range of 30 [18].

Studies have found that the sensitivity of SLN mapping is higher than that of full PLN dissection [19]. This can be explained by the detection of uncommon draining pathways (e.g., common iliac, presacral or para-aortic), which are not routinely included in systematic PLN dissection, complete SLN excision and the routine use of ultrastaging [20]. Data in the literature regarding the diagnostic value of SLNB varies greatly among different series, with sensitivity ranging from 33.3% to 100% [21, 22]. The main cause of the insufficient sensitivity was its inability to detect low volume metastatic disease (LVD): micrometastases and ITCs [23]. In our study, the results of anti-keratinose immuno-histochemical staining indicated that no LVDs were missed in the SLNs; the results that used frozen sections were identical to those that used permanent staining. Even so, we are of the opinion that the failure to detect LVD constitutes an important limitation of SLNB in clinical management. Micrometastases in cervical cancer have been reported to have a negative prognostic impact; they compromise the recurrence rate [24] and progression free survival [25]. They have also been found to be an independent prognostic factor for survival in a recent study. A 23% reduction in overall survival was reported in the patients with micrometastases as compared to those with negative nodes [19, 26]. It is necessary to strive for improvement regarding the methods of intra-operative assessment for the detection of micrometastasis. It appears that ITCs have no clinical significance, and their finding on definitive examination does not require modification of the therapeutic strategy [5, 27].

The abdominal approach for patients with tumors >2 cm in size permits a wider resection of the parametria and thus has the potential for the resection of larger tumors [28]. Wethington et al. [28] reported on 29 patients with tumors >2 cm in size, 22 of whom underwent ART. After a median follow-up time of 44 (range, 1–90) months, the authors reported no relapse or death. Litner et al. [29] followed up with 45 stage I cervical cancer patients with tumors >2 cm in diameter. Their 5-year survival rate (93.5%) was equal if not better than rates reported for patients treated by radical hysterectomies. Of the 42 patients who underwent definitive ART in the present study, two (4.8%) relapsed and one died from tumor progression. The other 40 patients achieved disease-free survival during a median follow-up time of 61 months. All of the data on survival seemed to support the feasibility and safety of fertility-sparing ART for selected patients with stage IB1 disease and tumors >2 cm in size.

There is a tremendous variation of recurrence rates among patients with tumors >2 cm in size, ranging from 0% to 29% [28–31]. These differences could be attributable to variations in patient population and sample sizes, follow-up periods, degrees of surgical radicality and the accuracy of intraoperative and preoperative selection of high-risk cases. In a previous study, we reported on 86 patients with stage IA2 to IB1 cervical cancer who underwent ART. The recurrence rates of these patients after a median follow-up of 77 (range, 3–142) months was 4.4% (2/45) for tumors ≤2 cm in size, and 4.9% (2/41) for tumors >2 cm in size, which was not significantly different (*P* = 1). This suggests that tumors >2 cm in size are not a risk factor for oncologic outcomes after ART. In the present study, of the two patients with recurrence, one had poor tumor differentiation and deep stromal invasion (DSI) <1/2, and the other had poor tumor differentiation, DSI <1/2 and or lymphovascular space invasion (LVSI). Although the patient sample size was too small to provide statistically reliable data, these results are consistent with LVSI and poor tumor differentiation being important risk factors for recurrence.

In our series, of 19 patients who attempted to conceive, five (26.3%) became pregnant spontaneously. Three patients had first semester miscarriages, one patient had a second trimester miscarriage, and one patient had a live birth in the third trimester. There are several factors that can further affect fertility after ART including a higher risk of adhesions after the abdominal approach, a greater disruption of pelvic nerve innervations to the uterus, and abnormality of the fallopian tubes caused by larger resection of the paracervix tissue. Various approaches have been suggested in several case series for preventing preterm birth in women who experienced radical trachelectomies. Reproductive infertility specialists were also reported to be important in helping patients conceive. Unfortunately, there is limited data available about preterm delivery and there are no definite guidelines on the topic [32, 33].

In conclusion, although we embrace and endorse ART with SLNB as a feasible procedure for patients with IB1 cervical cancer and tumors >2 cm in size, further research is needed. New molecular techniques that offer preoperative assessment of SLNs are especially interesting. Strategies should be developed for the prevention of preterm birth in women who have undergone radical trachelectomies. Data regarding long-term safety, fertility, and neonatal outcome for this fertility-preserving concept are still needed.

## MATERIALS AND METHODS

### Patients

Patients diagnosed with stage IB1 (Figure 2009) cervical cancer with tumors >2 cm, who were treated at the Shandong Cancer Hospital and Institute between March 2003 and July 2015, were recruited into this study. Patients who met the institutional eligibility criteria (Table 5) were considered eligible to undergo ART with pelvic lymphadenectomy, with or without para-aortic lymphadenectomy. The study was approved by our institutional review board. All patients enrolled in the study provided written informed consent before surgery.

**Table 5:** Suggested clinical eligibility criteria for ART and transperitoneal pelvic lymphadenectomy

Criteria
1. Histologic diagnosis of squamous cell carcinoma, adenocarcinoma, or adenosquamous carcinoma
2. FIGO stage IB1 disease with tumors > 2 cm
3. Age ≤ 40 years
4. Desire to preserve fertility
5. No clinical evidence of impaired fertility
6. Preoperative magnetic resonance imaging (MRI) of pelvis and abdomen, or appropriate imaging protocol with no evidence of pelvic lymph node metastases, and confirmation of tumor limited to the cervix

### SLN detection and surgical technique

SLNs were detected by means of an isotope injection into the uterine cervix. On the day before surgery, we injected fluid containing 2.5 ml (100.0 MBq) of 99mTc-labeled sulfur colloid submucosally into four quadrants of the cervix (3, 6, 9 and 12 o’clock positions). After systematic exploration of the peritoneal cavity, the SLNs were detected with a handheld gamma probe (Neoprobe, neo2000TM: AR-MED Ltd.) that was used to fully scan the pelvic sidewall, presacral area and para-aortic lymph beds. SLNs were identified based on the presence of radioactive hot nodes measuring >5-fold above the background level. The SLNs were then excised with safety margins and submitted for fast-frozen sectioning (adenocarcinoma patients received bilateral ovarian biopsies at this time). After removing the SLNs, patients routinely received bilateral pelvic lymphadenectomy to ensure the absence of PLN metastasis by analysis of frozen sections. If SLNs were identified in aberrant locations para-aortic lymphadenectomy was also conducted.

Intraoperative frozen section analysis of the SLNs was performed to guide the selection of surgical treatment. ART was performed if the SLNs and bilateral ovarian biopsies were negative. The round ligaments were transected and ligated laterally. Then paravesicle and pararectal spaces were developed with ligation of uterine arteries and preservation of ovarian vessels. The ureter was mobilized from the medial sheath of the broad ligament to the level of the cardinal ligament. A vessel loop was used to place traction on the ureter. Intraoperatively, the cervical canal was separated from the corpus uteri and opened through a longitudinal section at the 12 o’clock location. A frozen section was prepared 10 mm from the surgical margin to ensure a 10-mm negative endocervical margin. If frozen sections suggested tumor-free surgical margins, the uterus was reconstructed to the upper vagina with absorbable sutures. If the frozen section of the SLNs, bilateral ovarian sample and/or surgical margin was cancerous, radical hysterectomy and/or para-aortic lymphadenectomy was performed. After the incision was taken down and the specimen was removed, a cerclage suture (non-absorbable no. 10 silk suture) was performed in patients who underwent ART.

Surgical specimens were examined using routine hematoxylin and eosin staining. All SLNs were immuno-stained with anti-cytokeratin antibody to detect micrometastasis. The intensity of protein expression was evaluated using OPTIMAS 6.5 software.

### Adjuvant therapy

Recommendations for postoperative chemotherapy or radiation therapy were based on high-risk pathological features, such as nodal metastasis or parametrial involvement, as well as for those with intermediate risk factors, such as adenocarcinoma, poor differentiation, DSI or LVSI. Specific treatment recommendations were at the discretion of the gynecological oncologist and radiation oncologist. Adjuvant chemotherapy included the following: a combination of cisplatin, vincristine and bleomycin (combination PVB); a combination of cisplatin, etoposide and bleomycin (combination PEB); or a combination of paclitaxel and carboplatin (combination TC). The radiation therapy regimen consisted of the following: external beam radiation planned using an ADAC Treatment Planning System; delivered using 15 MV X-rays from a Varian 21EX (Palo Alto, CA, USA); and designed based on CT scans. External irradiation was delivered in four fractions per week (2.0 Gy daily fractions) to a total dose of 50–55 Gy.

### Follow-up

After surgery, all patients were followed up at regular intervals with clinical examinations, trans-vaginal ultrasound, Papanicolaou tests and squamous cell carcinoma antigen tests; combined oral contraceptives were recommended for sexually active patients. Survival, recurrence, pregnancy and childbearing information were obtained from personal contact with the patient or her family. Overall survival was calculated from the date of diagnosis. Surviving patients were censored on the date of last follow-up.

### Statistical analysis

Continuous variables were compared using Student's *t* test; categorical variables were compared using the chi squared test or Fisher's exact test, as appropriate. A *P* value < 0.05 was considered significant. All analyses were performed using SPSS version 19.0 (SPSS Inc., Chicago, IL, USA).
